# Combining Participatory Influenza Surveillance with Modeling and Forecasting: Three Alternative Approaches

**DOI:** 10.2196/publichealth.7344

**Published:** 2017-11-01

**Authors:** John S Brownstein, Shuyu Chu, Achla Marathe, Madhav V Marathe, Andre T Nguyen, Daniela Paolotti, Nicola Perra, Daniela Perrotta, Mauricio Santillana, Samarth Swarup, Michele Tizzoni, Alessandro Vespignani, Anil Kumar S Vullikanti, Mandy L Wilson, Qian Zhang

**Affiliations:** ^1^ Computational Health Informatics Program Boston Children's Hospital Boston, MA United States; ^2^ Computational Epidemiology Group Division of Emergency Medicine Boston Children’s Hospital Boston, MA United States; ^3^ Harvard Medical School Boston, MA United States; ^4^ Network Dynamics and Simulation Science Laboratory Biocomplexity Institute Virginia Tech Blacksburg, VA United States; ^5^ Booz Allen Hamilton Boston, MA United States; ^6^ Computational Epidemiology Laboratory Institute for Scientific Interchange Turin Italy; ^7^ Centre for Business Networks Analysis University of Greenwich London United Kingdom; ^8^ Laboratory for the Modeling of Biological and Socio-technical Systems Northeastern University Boston, MA United States

**Keywords:** forecasting, disease surveillance, crowdsourcing, nonresponse bias

## Abstract

**Background:**

Influenza outbreaks affect millions of people every year and its surveillance is usually carried out in developed countries through a network of sentinel doctors who report the weekly number of Influenza-like Illness cases observed among the visited patients. Monitoring and forecasting the evolution of these outbreaks supports decision makers in designing effective interventions and allocating resources to mitigate their impact.

**Objective:**

Describe the existing participatory surveillance approaches that have been used for modeling and forecasting of the seasonal influenza epidemic, and how they can help strengthen real-time epidemic science and provide a more rigorous understanding of epidemic conditions.

**Methods:**

We describe three different participatory surveillance systems, WISDM (Widely Internet Sourced Distributed Monitoring), Influenzanet and Flu Near You (FNY), and show how modeling and simulation can be or has been combined with participatory disease surveillance to: i) measure the non-response bias in a participatory surveillance sample using WISDM; and ii) nowcast and forecast influenza activity in different parts of the world (using Influenzanet and Flu Near You).

**Results:**

WISDM-based results measure the participatory and sample bias for three epidemic metrics i.e. attack rate, peak infection rate, and time-to-peak, and find the participatory bias to be the largest component of the total bias. The Influenzanet platform shows that digital participatory surveillance data combined with a realistic data-driven epidemiological model can provide both short-term and long-term forecasts of epidemic intensities, and the ground truth data lie within the 95 percent confidence intervals for most weeks. The statistical accuracy of the ensemble forecasts increase as the season progresses. The Flu Near You platform shows that participatory surveillance data provide accurate short-term flu activity forecasts and influenza activity predictions. The correlation of the HealthMap Flu Trends estimates with the observed CDC ILI rates is 0.99 for 2013-2015. Additional data sources lead to an error reduction of about 40% when compared to the estimates of the model that only incorporates CDC historical information.

**Conclusions:**

While the advantages of participatory surveillance, compared to traditional surveillance, include its timeliness, lower costs, and broader reach, it is limited by a lack of control over the characteristics of the population sample. Modeling and simulation can help overcome this limitation as well as provide real-time and long-term forecasting of influenza activity in data-poor parts of the world.

## Introduction

Epidemiological surveillance is an important facet in the detection and prevention of the spread of an epidemic [1]. Knowing which diseases and variations of these diseases are present can help medical researchers identify appropriate interventions as well as strategies for treatment to reduce overall impact of the disease, including mortality. Because of the utility of such data, a number of agencies collect and distribute surveillance reports on prevailing epidemics or other diseases of interest. In the United States, the Centers for Disease Control and Prevention (CDC) produces surveillance counts for influenza and other diseases based on reports from state and local laboratories and medical health centers (www.cdc.gov/flu/weekly/summary.htm). Internationally, the World Health Organization and other agencies produce surveillance data for a number of emerging diseases such as Zika and Ebola (www.who.int/emergencies/zika-virus/ situation-report/25-august-2016/en/).

While these clinically-based disease surveillance systems are necessary to keep track of disease prevalence and contain their spread, they have practical limitations [2]. Given the time required to collate surveillance numbers, the reports are usually several weeks old, resulting in a mismatch between the public health response and conditions on the ground [3]. Depending upon the transmissibility of the epidemic, there can be a big difference in prevalence from week to week. Additionally, even when collecting data from local medical centers, coverage is not always uniform. As a result, the CDC weights the public health response based on state population as well as a region’s past history of influenza-like illness (ILI) cases [1]. Finally, the level of detail afforded by the medical laboratories and centers reporting to these clinically-based systems may not be sufficient for examining the type of regional demographics that help to identify interventions that are likely to be effective [3].

A number of algorithms and technical approaches have been developed in recent years to attempt to mitigate the shortcomings in clinically collected surveillance data. To address the time delay between when surveillance data become available and the current date, approaches have been developed for ILI that use mechanistic modeling based on epidemiological knowledge of the pathways of flu transmission to estimate near real-time and future estimates of flu activity [4,5]. Other approaches have attempted to leverage information from constantly changing Internet-based data sources to identify patterns that may signal a change in the incidence of ILI cases in a population. These data sources include Internet search engines [6-12], Twitter and its microblogs [13-17], clinicians’ Internet search engines [18], and participatory disease surveillance systems where responders on the ground report on disease propagation [19]. Sharpe et al [20] conducted a comparative study to analyze whether Google-, Twitter-, or Wikipedia-based surveillance performs the best when compared to CDC ILI data.

In addition to helping address the time delay problem, participatory disease surveillance can also offer valuable insight into the characteristics of a disease and the demographics of the affected population [19,21-24]. It can help to augment coverage in areas where there are fewer medical centers or where infected people are less likely to go for clinical evaluation. Finally, participatory surveillance also offers a good opportunity to promote awareness of an epidemic [25].

Participatory surveillance has its limitations as well, especially participatory bias resulting from nonuniform coverage and from waning interest and participation over the duration of an epidemic [22]. Additionally, although not addressed with the examples in this paper, training and trust issues may lead to under- or incorrect reporting [23]. Combining participatory surveillance with modeling and simulation can not only help to reduce participatory bias but can also improve real-time forecasting and thus help identify which interventions are most likely to be effective over time in a given area.

In this article, we investigate how an understanding of the results from 3 participatory disease surveillance systems, WISDM (Widely Internet-Sourced Distributed Monitoring), Influenzanet, and Flu Near You (FNY), can be or have been extended through the use of modeling, simulation, and forecasting.

## Methods

### Widely Internet-Sourced Distributed Monitoring and Synthetic Information

#### Using Modeling to Measure Participatory Bias

WISDM is a Web-based tool developed at Virginia Tech that supports crowdsourced behavioral data collection, inspection, and forecasting of social dynamics in a population. When integrated with online crowdsourcing services such as Amazon’s Mechanical Turk (MTurk), WISDM provides a cost-effective approach to real-time surveillance of potentially evolving disease outbreaks [26]. So far, WISDM has been used primarily to collect demographic and health behavior data for epidemiological research. Here, we describe how modeling can be used in combination with WISDM to measure participatory (nonresponse) bias.

Crowdsourcing platforms like MTurk can be used to recruit responders for a low fee. MTurk allows requesters to recruit human intelligence to conduct tasks that computers cannot do; individuals who browse among existing jobs are called workers. However, there is some concern that users recruited on crowdsourcing platforms may not be representative of the population at large [27,28]. MTurk workers tend to be young, educated, and digitally savvy, so their responses may systematically differ from the responses of those who did not participate in the survey. Given this potential for nonresponse or participatory bias, understanding how to use data from such surveys for epidemic surveillance is a challenge.

**Figure 1:**
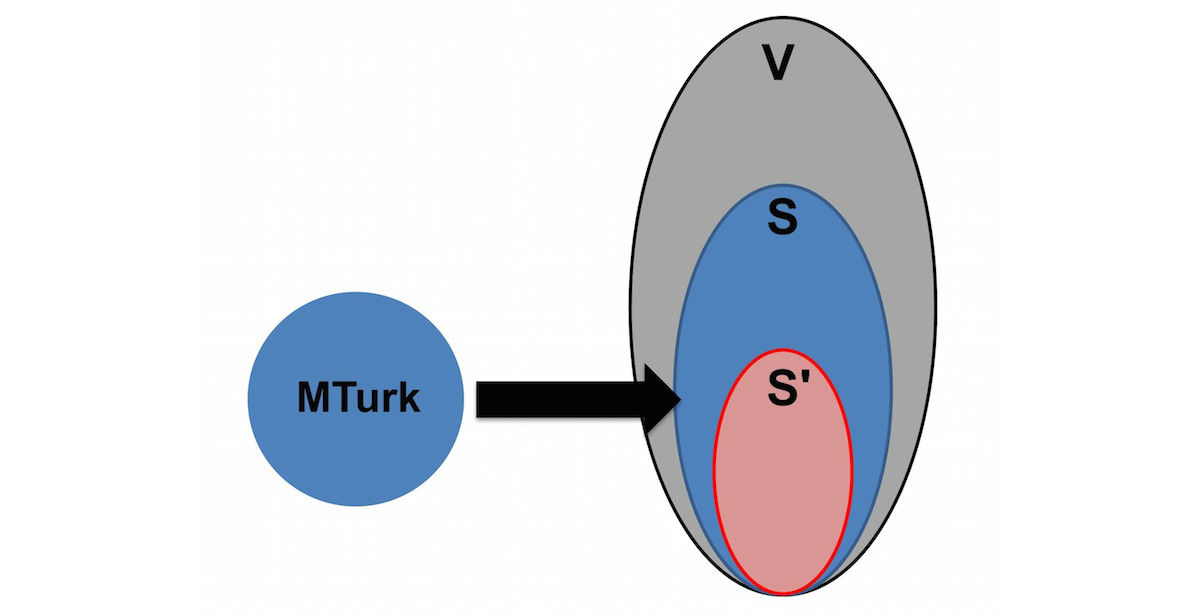
Mapping of MTurk sample to synthetic individuals.

To address this issue, we developed a simulation-based approach. Specifically, we combined results of a survey of Delhi, India, residents conducted on WISDM through MTurk with agent-based simulations of the Delhi population to understand the MTurk sample bias. First, we constructed a synthetic population that was statistically indistinguishable from the Delhi census (V in Figure 1), thus providing the best extant at-scale representation of the population.

The synthetic population is generated by combining marginal distributions of age, household income, and household size for each Census block group with the corresponding Public Use Microdata Sample. This is done using the iterative proportional fitting procedure [29]. Validation is done by comparing distributions of variables not included in the iterative proportional fitting step with the corresponding distributions in the generated synthetic population. The procedure is guaranteed to converge [30] and the inferred joint distribution is the best in the maximum entropy sense [31].

The synthetic population is generated for each block group, which is the highest resolution at which US Census data are available publicly. We generate social contact networks (contact matrices) for the synthetic population through a detailed data-driven model where, after the agents matching the region's demographics are generated, they are assigned home locations using road network data (from Here, formerly known as Navteq), daily activity patterns are assigned using the National Household Travel Survey data, and activity locations are assigned using Dun and Bradstreet data. This allows social contact networks to be extracted based on agents being simultaneously present at locations for overlapping durations. We refer to the literature for a detailed description of the construction of synthetic populations and their applications [32-41].

­­­From this synthetic population, we selected individuals whose demographics most closely matched the demographics of the MTurk respondents of the WISDM survey (the S in Figure 1). Then, epidemic characteristics of this selected subsample were studied and compared to the epidemic characteristics of the entire synthetic population.

#### Process for Finding the Mechanical Turk–Matched Delhi Synthetic Population

First, we used WISDM to collect demographics and health behaviors of about 600 MTurk workers; the health behaviors included preventative and treatment behaviors related to influenza. Then we calculated the Euclidean distance between each of these approximately 600 responders and every person in the synthetic population of the same age, gender, and household size. Next, we selected the closest synthetic matches to each survey respondent. If more than 1 match was identified, all of the matches were retained. We repeated this procedure for each responder in the survey, which provided us with a subpopulation of the synthetic population that most closely matched the WISDM-based survey respondents. This subpopulation is denoted by S in Figure 1, and V denotes the total synthetic population of Delhi.

However, the synthetic subpopulation (S) was not statistically representative of the MTurk sample given that survey respondents could be matched with multiple individuals. Thus, we used stratified sampling to construct a finer sample of the synthetic population that was equivalent to those who took the MTurk survey.

Specifically, we divided both the survey and synthetic subpopulation (S) data into H mutually exclusive strata, where each stratum corresponded to a unique combination of 3 demographic variables, specifically age, gender, and household size. Only these 3 demographic factors were used for stratification since India Census did not have information on other common socioeconomic variables like income, education, employment, and access to Internet. Variables such as income and access to Internet could be especially important in matching MTurk with individuals in the synthetic population, but due to lack of data this could not be done. This is a significant limitation of the current analysis which we expect to improve upon as more data becomes available in the future.

We discretized age into A distinct intervals and household size into B intervals. Gender was split into 2 groups. This resulted in H=2AB strata. Because all matched synthetic people had been retained, the number of observations (N_1_) in the synthetic subpopulation (ie, first stratum of subpopulation S) was much larger than the number of observations (n_1_) in the first stratum in the MTurk survey (ie, first stratum of the actual survey sample). Thus, to obtain a representative sample of this first stratum, n_1_ observations were randomly sampled from the synthetic subpopulation without replacement. The same procedure was performed for all the remaining strata. This provided us with the final MTurk-matched Delhi synthetic population sample set S' in Figure 1, which demographically matched the MTurk survey data.

#### Comparing Epidemic Outcomes Using Widely Internet-Sourced Distributed Monitoring

Our goal was to understand the differences in influenza epidemic outcomes across the 3 populations (V, S, and S’). We considered 3 different metrics for measuring epidemics: (1) the size of the epidemic (ie, the attack rate), (2) the peak number of infections, and (3) the time it takes for the epidemic to peak. A difference in these metrics between S and S' would be equivalent to the sample bias if we assume S captures the entire MTurk population. This may not be true unless the sample size is very large, which is not the case in this study. However, for very large samples, it would give the sample bias since S' is the sample and S is the entire synthetic subpopulation that matches the attributes of the sample. Differences between V and S metrics would be equivalent to the nonresponse bias because individuals outside S did not participate in the survey.

In order to compare the epidemic outcomes, we simulated an influenza outbreak using a susceptible, exposed, infected, and recovered (SEIR) disease model [34,35] in the synthetic Delhi population. Each node in the network represents an individual, and each edge represents a contact on which the disease can spread. Each node is in 1 of 4 states at any given time: S, E, I, or R. An infectious person spreads the disease to each susceptible neighbor independently with a probability referred to as the transmission probability, given by p=λ(1–(1–τ)^Δt^), where λ is a scaling factor to lower the probability (eg, in the case of vaccination), τ is the transmissibility, and Δt is the duration of interaction in minutes. Durations of contact are labels on the network edges. A susceptible person undergoes independent trials from all of its neighbors who are infectious. If an infectious person infects a susceptible person, the susceptible person transitions to the exposed (or incubating) state. The exposed person has contracted influenza but cannot yet spread it to others. The incubation period is assigned per person according to the following distribution: 1 day (30%), 2 days (50%), 3 days (20%). At the end of the exposed or incubation period, the person switches to an infected state. The duration of infectiousness is assigned per person according to the following distribution: 3 days (30%), 4 days (40%), 5 days (20%), 6 days (10%). After the infectious period, the person recovers and stays healthy for the simulation period. This sequence of state transitions is irreversible and is the only possible disease progression. We seed the epidemic in a susceptible population with 10 infections that are randomly chosen every day. A total of 25 replicates were run to account for the stochastic randomness arising from the selection of initial infectors.

### Influenzanet

In 2008, a large research project funded by the European Commission and coordinated by the Institute for Scientific Interchange in Turin, Italy, led to the creation of Influenzanet, a network of Web-based platforms for participatory surveillance of ILI in 10 European countries [42]. The ambition was to collect real-time information on population health through the activity of volunteers who provide self-reports about their health status and, by combining this real-time data feed with a dynamical model for spatial epidemic spreading, build a computational platform for epidemic research and data sharing. The results of this multiannual activity have been used to create a novel, modular framework (the FluOutlook framework) capable of capturing the disease transmission dynamics across country boundaries, estimating key epidemiological parameters, and forecasting the long-term trend of seasonal influenza [43].

**Figure 2:**
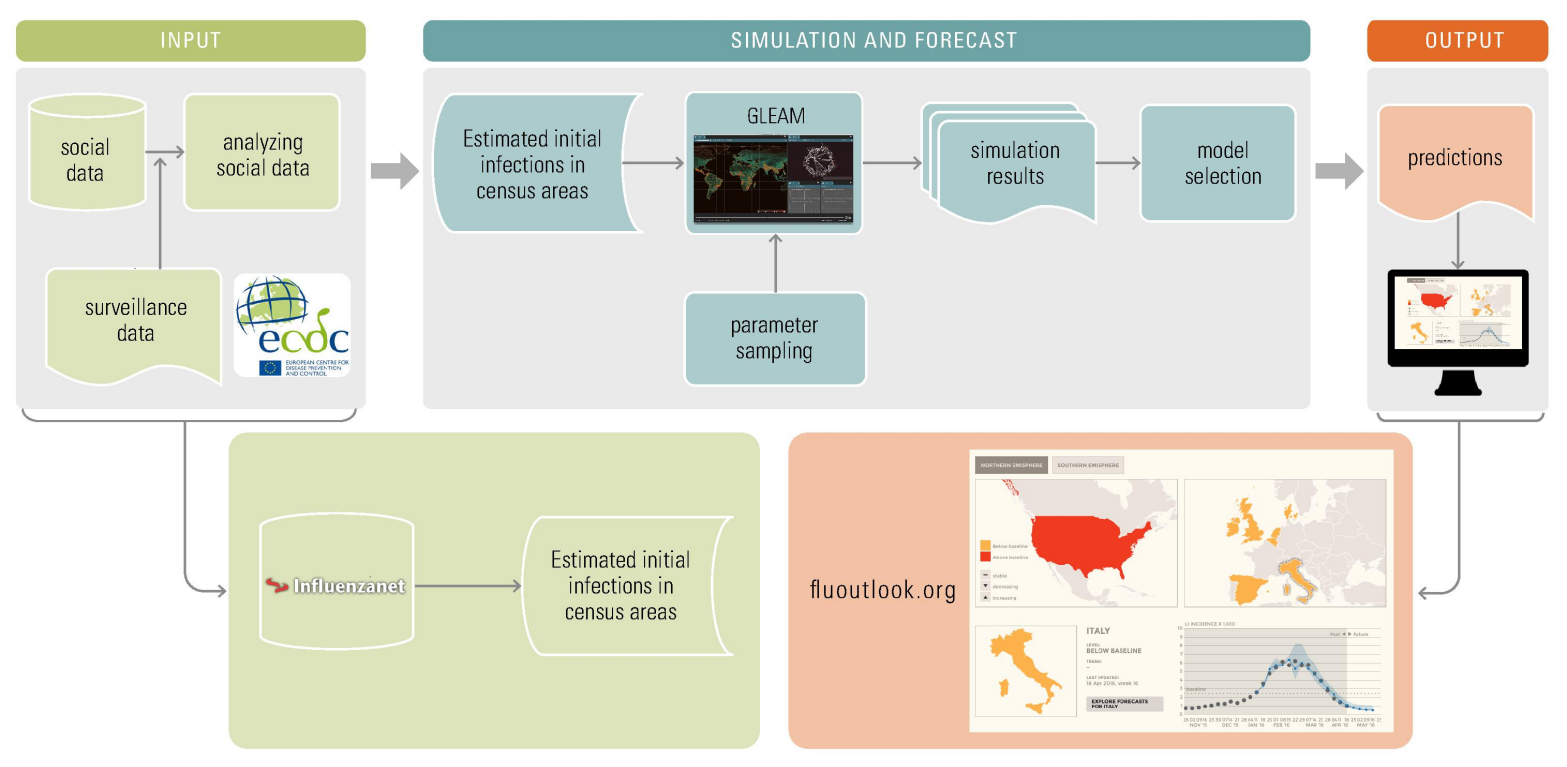
The FluOutlook framework.

The framework consists of 3 main components: (1) input, (2) simulation and forecast, and (3) output (Figure 2).

The input component estimates initial infections for a given week in any census area from collected self-reported information from volunteers on Influenzanet platforms or from other data proxies like Twitter. Influenzanet data collection has been described in several previous papers [44]. The number of users reporting a case of ILI each week is used to calculate the weekly incidence of ILI among active users. Active users are those who completed at least 1 Influenzanet symptoms questionnaire during the influenza season. Since users report their place of residence at the level of postal codes, the ILI weekly incidence can be calculated at the resolution level of postal codes.

The simulation and forecast component is a computational modeling and simulation engine named Global Epidemic And Mobility model (GLEAM) [45,46]. The GLEAM dynamical model is based on geographical census areas defined around transportation hubs and connected by long- and short-range mobility networks. The resulting meta-population network model can be used to simulate infectious disease spreading in a fully stochastic fashion. The simulations, given proper initial conditions and disease model, generate an ensemble of possible epidemic evolution for epidemic parameters such as newly generated cases. In the application to seasonal influenza, GLEAM is limited to the level of a single country with only the population and mobility of the country of interest taken into account. The number of ILI cases extracted from the Influenzanet platforms are mapped onto the corresponding GLEAM geographical census areas and used as seeds to initialize the simulations. The unique advantage provided by using the data collected by the Influenzanet platform as initial conditions consist in the high resolution, in time (daily) and space (postal code level), with which data are available. This geographical and temporal resolution for the initial conditions cannot be achieved with any other signal. Moreover, these are not proxy data for the ILI activity among the population but indeed represent a high-specificity ground truth for the initial conditions that cannot be obtained with any other source of information. Given these high quality and highly reliable initial conditions, the GLEAM simulations perform a Latin hypercube sampling of a parameter space covering possible ranges of transmissibility, infection periods, immunization rates, and a tuning parameter regulating the number of generated infected individuals. In the prediction component of the framework, the large-scale simulations generate a statistical ensemble of the epidemic profiles for each sampled point in the parameter space. From each statistical ensemble, the prediction component measures its likelihood function with respect to up-to-date ILI surveillance data and selects a set of models by considering a relative likelihood region [47].

The set of selected models represents the output component and provides both long-term (ie, 4 weeks in advance) and short-term predictions for epidemic peak time and intensity. Results are disseminated as interactive plots that can be explored on the public website fluoutlook.org [48].

To quantify the simulation’s forecast performance, the Pearson correlation between each predicted time series and sentinel doctors’ surveillance time series can be used. Moreover, the mean absolute percent error can be used to evaluate the magnitude estimation and the peak week accuracy defined as the percentage of the selected ensemble of simulations providing predictions within 1 week for peak time.

### Flu Near You

FNY is a participatory disease surveillance system launched in October 2011 by HealthMap of Boston Children’s Hospital, the American Public Health Association, and the Skoll Global Threats Fund [17]. FNY maintains a website and mobile app that allows volunteers in the United States and Canada to report their health information using a brief weekly survey. Every Monday, FNY sends users a weekly email asking them to report whether or not they experienced any of the following symptoms during the previous week: fever, cough, sore throat, shortness of breath, chills, fatigue, nausea, diarrhea, headache, or body aches. Users are also asked to provide the date of symptom onset for any reported symptoms. Users experiencing fever plus cough and/or sore throat are considered by FNY to be experiencing an ILI. FNY’s definition of ILI differs slightly from the US CDC outpatient Influenza-Like Illness Surveillance Network (ILINet) definition, which defines ILI as fever plus cough and/or sore throat without a known cause other than influenza.

**Figure 3:**
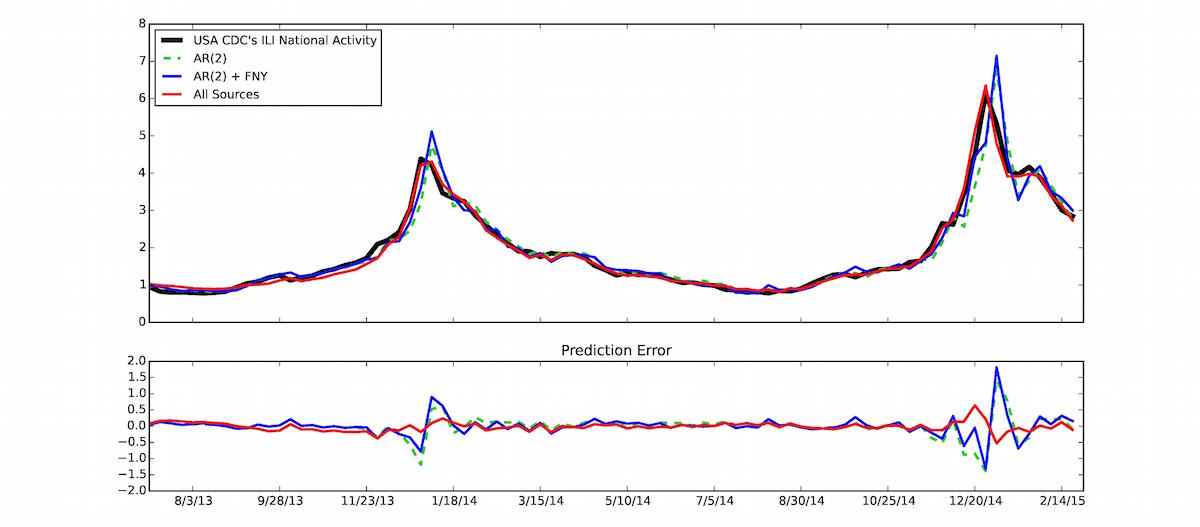
(Top panel) The US Centers for Disease Control and Prevention (CDC) influenza-like illness (ILI) percent value (y-axis) is displayed as a function of time (x-axis). Predictions produced 1 week ahead of the publication of CDC-ILI reports using (1) only historical CDC information via an autoregressive model, AR(2), (2) an autoregressive model that combines historical CDC information with Flu Near You (FNY) information, AR(2)+FNY, and (3) an ensemble method that combines multiple data sources including FNY, Google search frequencies, electronic health records, and historical CDC information (all sources) are shown. (Bottom panel) The errors between the predictions and the CDC-reported ILI for each prediction model are displayed.

FNY was conceived to capture flu activity in a population group that may not necessarily seek medical attention, while CDC’s ILINet was designed to monitor the percentage of the population seeking medical attention with ILI symptoms. Recent estimates confirm that only approximately 35% of FNY participants who report experiencing ILI symptoms seek medical attention. Despite this design (and observed) difference and because these 2 distinct groups (those seeking medical attention versus those not doing so) interact, large changes in ILI in the CDC’s ILINet are also generally observed in the FNY signal, as shown in Figure 3 for the 2013-2014 and 2014-2015 flu seasons and as previously shown by Smolinski et al [19]. To produce Figure 3, spikes of unrealistic increased FNY ILI rates (calculated as the weekly number of users who experienced ILI divided by the total number of reports received during the same week) were first removed. These unrealistic spikes (defined as a weekly change in the FNY ILI rates larger than 10 standard deviations from the mean change of the last 4 weeks) are often associated with media attention on FNY that causes a temporary surge of interest in the system among people sick with the flu, as described Aslam et al [17]. Flu estimates were then produced 1 week ahead of the publication of CDC reports by combining historical CDC-reported flu activity (via a lag-2 autoregressive model) with the smoothed weekly FNY rates. These flu estimates are displayed in blue and labeled AR(2)+FNY on Figure 3.

The reason why we used CDC-reported ILI rates as our reference for traditional flu surveillance is because these ILI rates have been recorded for multiple years, and public health officials have used them as proxies of influenza levels in the population. This is consistent with multiple influenza activity prediction studies in the United States [7-9,49-50]. With the intent of providing more timely yet still familiar information to public health officials, we use the smoothed FNY ILI rates as one of multiple data inputs into the HealthMap Flu Trends influenza surveillance and forecasting system [51].

The HealthMap Flu Trends system relies on a machine-learning modeling approach to predict flu activity using disparate data sources [49] including Google searches [8-9], Twitter [15], near real-time electronic health records [50], and data from participatory surveillance systems such as FNY [19]. The HealthMap Flu Trends system provides accurate real-time and forecast estimates of ILI rates at the national as well as regional levels in the United States up to 2 weeks ahead of CDC’s ILINet flu reports.

The multiple data sources entered into the HealthMap Flu Trends system are each individually processed using machine-learning algorithms to obtain a predictor of ILI activity. These individual predictions of ILI rates are then fed into an ensemble machine-learning algorithm that combines the individual predictions to produce robust and accurate ILI estimates, described by Santillana et al [49]. The estimates produced by this ensemble machine-learning approach outperform all of the predictions made using each of the data sources independently.

## Results

### Widely Internet-Sourced Distributed Monitoring–Based Results

**Figure 4:**
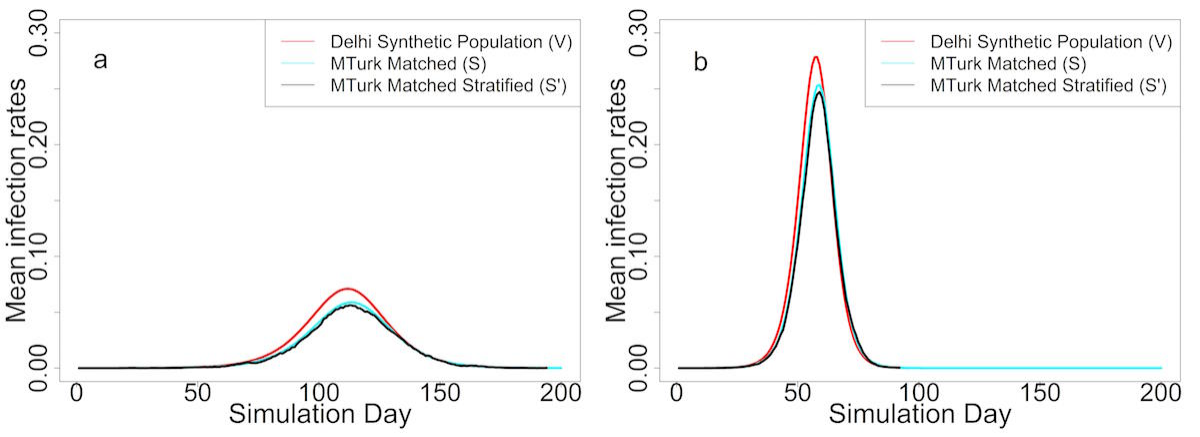
(a) Epidemic curves under low transmission rate. (b) Epidemic curves under high transmission rate.

The results based on WISDM are illustrated as time series of daily infections (also called epidemic curves) in Figure 4. Figures 4 a and 4 b correspond to low transmission (0.00003 per minute of contact time and R_0_=1.4) and high transmission (0.00006 per minute of contact time and R_0_=2.7) rates, respectively. The red epidemic curve in each represents the entire Delhi synthetic population (V). The black and blue epidemic curves show results for the MTurk-matched Delhi synthetic population (S') and the entire MTurk-matched Delhi synthetic population (S), respectively. Under a high transmission rate, the attack rate and peak infection rate are higher but the time-to-peak is lower. This is expected since a higher transmission rate spreads the disease quickly and to more individuals in the population.

If surveillance is restricted to only the MTurk sample (S'), the level of bias would equal the difference between the red and black curves. This difference represents a combination of the nonresponse bias (difference between the red curve and blue curve) and the sample-size bias (difference between the blue curve and black curve).

In order to measure the significance of the total bias, the nonresponse bias, and the sample-size bias of the simulation illustrated in Figure 4, we tested the differences in attack rate, peak infection rate, and time-to-peak by using the 2-sample *t* test. The mean difference, 95% confidence intervals, and *P* values are summarized in Tables 1 and 2 for low and high transmission rates, respectively.

As shown in Table 1, with a low transmission rate (0.00003), the attack rate for S' is about 10% lower than that for V, while the peak infection rate for S' is 1.36% lower and the epidemic curve peaks 1 day later. Total biases for all 3 metrics are statistically significant. Also for all 3 metrics, the nonresponse bias is larger than the sample bias and dominates the total bias. This is consistent with the fact that MTurk survey responders tend to be younger, educated males among whom the incidence of disease is typically lower than much of the rest of the population.

Results for the higher transmission rate (0.00006) are similar (Table 2). Note, however, that the difference between the red and black curves (in Figure 4) shrinks as the transmission rate becomes higher.

**Table 1:** Bias in epidemic metrics under low transmission rate.

Metric		Nonresponse bias (V-S)	Sample-size bias (S-S')	Total bias (V-S')
**Attack rate**				
	Mean difference, %	7.90	2.13	10.03
	95% CI	7.88 to 7.91	1.58 to 2.68	9.47 to 10.58
	*P* value	<.001	<.001	<.001
**Peak infection rate**				
	Mean difference, %	1.22	0.14	1.36
	95% CI	1.22 to 1.22	0.05 to 0.23	1.27 to 1.45
	*P* value	<.001	.003	<.001
**Time to peak**				
	Mean difference, days	–1.76	0.76	–1
	95% CI	–1.96 to –1.56	0.16 to 1.36	–1.58 to –0.42
	*P* value	<.001	.02	.002

**Table 2:** Bias in epidemic metrics under high transmission rate.

Metric		Nonresponse bias (V-S)	Sample-size bias (S-S')	Total bias (V-S')
**Attack rate**				
	Mean difference, %	6.31	3.58	9.90
	95% CI	6.30 to 6.32	3.06 to 4.10	9.38 to 10.42
	*P* value	<.001	<.001	<.001
**Peak infection rate**				
	Mean difference, %	2.51	0.63	3.14
	95% CI	2.50 to 2.53	0.49 to 0.77	3.01 to 3.28
	*P* value	<.001	<.001	<.001
**Time to peak**				
	Mean difference, days	–1.44	0.12	–1.32
	95% CI	–1.69 to –1.20	–0.10 to 0.34	–1.59 to –1.05
	*P* value	<.001	.28	<.001

### Influenzanet-Based Results

In this section, we show results for simulations and forecasts performed for the 2015-2016 influenza season. The input component of the framework has been initialized with ILI cases from a number of selected countries that are part of the Influenzanet network: Belgium, Denmark, Italy, the Netherlands, Spain, and the United Kingdom. In the simulation component, weekly surveillance data of sentinel doctors, also called traditional surveillance, in each of the selected countries have been used as ground truth to select the set of models with maximum likelihood.

**Figure 5:**
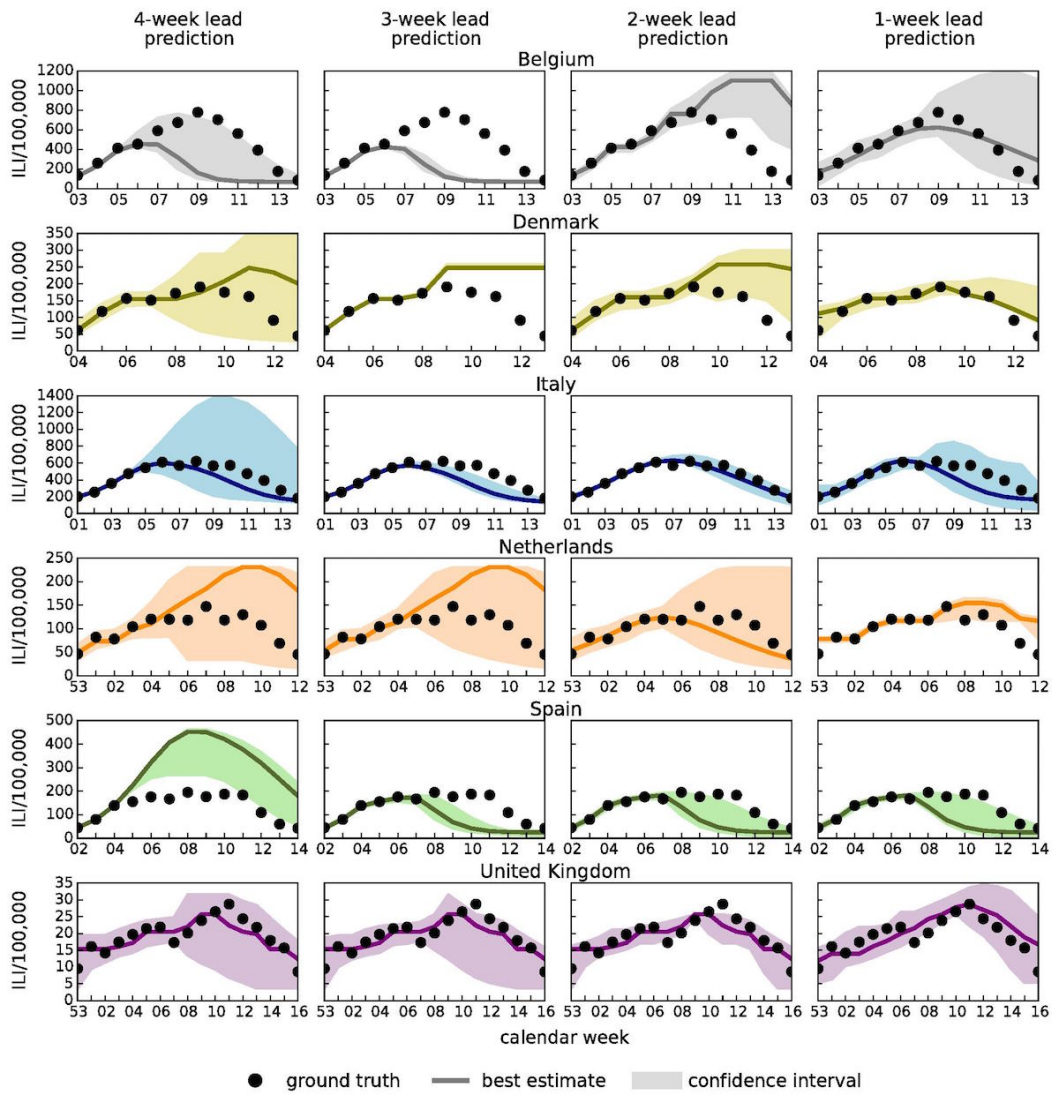
Epidemic profiles for Belgium, Denmark, Italy, the Netherlands, Spain, and the United Kingdom considering 4-week, 3-week, 2-week, and 1-week lead predictions. The best estimation (solid line) and the 95% confidence interval (colored area) are shown together with sentinel doctors' surveillance data (black dots) which represent the ground truth (ie, the target signals).

Figure 5 illustrates the results of 1-week, 2-week, 3-week, and 4-week predictions. We include results for 1-week, also called now-casting, predictions for the following reason. The now-casting predictions (ie, inferring the incidence value that the traditional influenza surveillance will report in the following week) are usually used to evaluate the performance of the predictions based on the model described in this work with respect to predictions based on linear regression models applied to traditional surveillance data only. In a recent work by Perrotta et al [52], it has been shown how real-time forecasts of seasonal influenza activity in Italy can be improved by integrating traditional surveillance data with data from the participatory surveillance platform called Influweb, and the now-casting predictions have been used as a benchmark test to compare the 2 approaches.

Figure 5 shows that for all countries under study, the empirical observations (ie, the ground truth of the traditional surveillance reference data represented as black dots in the figure) lie within the 95% confidence intervals for most weeks. This gives a qualitative indication of the accuracy of the predictions.

**Figure 6:**
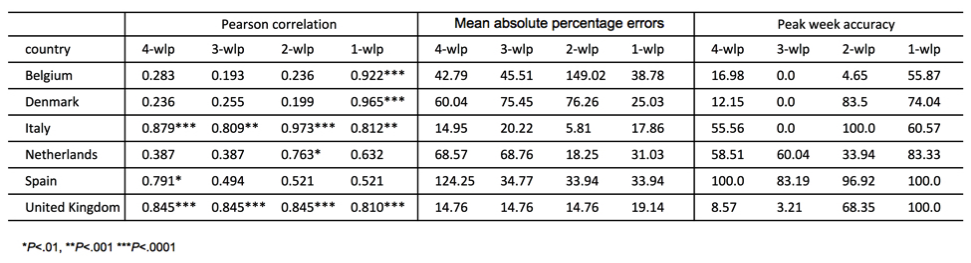
Pearson correlations, mean absolute percentage errors, and peak week accuracy obtained by comparing the forecast results and the sentinel doctors' influenza-like illness surveillance data along the entire season in each country.

In Figure 6, we show results for the Pearson correlation between each predicted time series and sentinel doctors' surveillance time series and also results for the mean absolute percent error (MAPE). As expected, the statistical accuracy of the ensemble forecasts increase as the season progresses. In the case of a 1-week lead prediction, the correlation is close to 1 for Italy and Belgium. The correlations are around 0.8 for 2-week predictions for the United Kingdom, around 0.7 for the Netherlands, and above 0.8 for 4-week lead predictions for United Kingdom and Italy. The peak magnitude is 1 of the free parameters we fit in the model. As the correlation increases as the season progresses, the MAPE (ie, the percentage error on the peak magnitude estimated by the model) decreases or remains quite stable for countries like the United Kingdom, in which the correlation is consistently high. For other countries, the performance is not as good and the peak magnitude is not so well estimated. Belgium and Spain are the 2 countries in which the performance is the worst. This might be due to the fact that the ILI incidence curve from Influenzanet in Spain is very noisy, mainly due to low participation, and this has affected the quality of the predictions in terms of amplitude and correlation. In Belgium, the ILI incidence data from traditional surveillance have been very noisy due to an unusually mild influenza season in this country. More information about the Influenzanet ILI incidence curves in the various countries can be found at the Influenzanet page (www.influenzanet.eu/ en/flu-activity/). The peak week accuracy also increases as the season progresses and, notably, accuracy is already above 60% with up to 4 weeks lead time in the case of Italy, the Netherlands, and Spain.

Overall, even for a peculiar influenza season such as 2015-2016, with an unusually late peak, the results show that our framework is capable of providing accurate short-range (1-week, 2-week) forecasts and reasonably accurate longer range (3-week, 4-week) predictions of seasonal influenza intensities and temporal trends.

**Figure 7:**
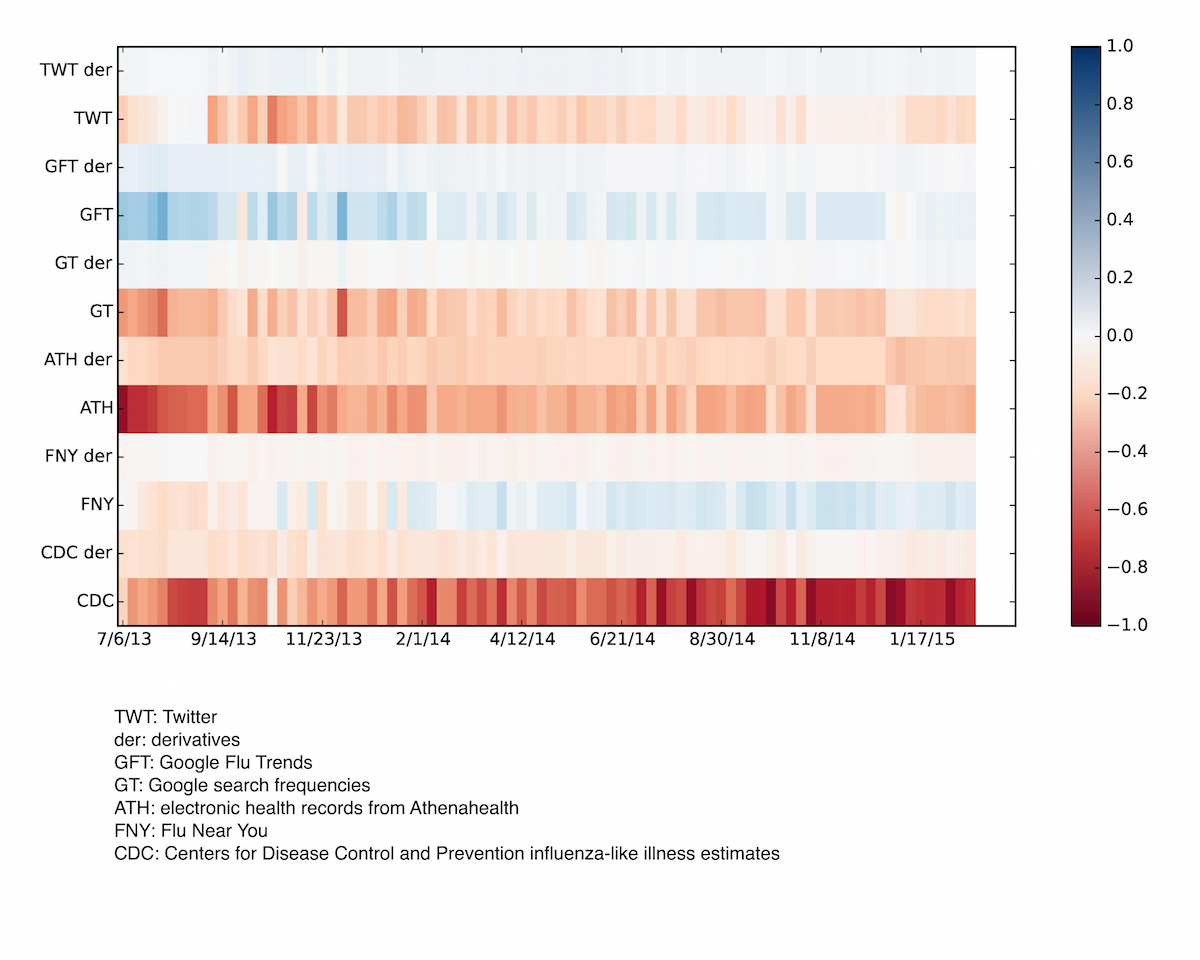
Heatmap showing the relevance of each of the input data sources on the flu prediction during the 7/2013-4/2015 time window (x-axis). These values change from week to week due to a dynamic model recalibration process. The multiple data sources entered into the HealthMap Flu Trends system are on the y-axis with their tendencies, or derivatives. The bar on the right is a color code of the magnitude of the regression coefficients of the multiple data sources used as inputs.

### Flu Near You–Based Results

We quantitatively confirmed that incorporating data from our participatory surveillance system improved real-time influenza predictions by comparing the aforementioned influenza estimates with estimates produced using a model based only on historical CDC-reported influenza activity (a lag-2 autoregressive model), labeled AR(2) in Figure 3. The correlation between the observed influenza activity and the estimates obtained using a model based only on historical ILI information for the 2013-2015 time window was 0.95, whereas the correlation with the model that incorporates FNY information was 0.96. While this represents a mild improvement in the correlation values, a more statistically robust test introduced by Yang et al [9] showed that the incorporation of FNY information led to a 10% mean error reduction (90% CI 0.04 to 0.24) when compared to the baseline autoregressive model. The bottom panel of Figure 3 shows visually the errors from each model.

HealthMap Flu Trends national-level real-time predictions that were available 1 week ahead of the publication of the weekly CDC reports for the 2013-2014 and 2014-2015 influenza seasons are shown in red on Figure 3. For comparison purposes, the correlation of the HealthMap Flu Trends estimates with the observed CDC ILI rates is 0.99 for the 2013-2015 time window, and the addition of multiple data sources leads to a mean error reduction of about 83% (90% CI 0.69 to 0.85) when compared to the estimates of the model that only uses CDC historical information (AR(2)). In Figure 7, the historical contributions of the different individual predictors (and their tendencies) in the HealthMap influenza estimates are displayed. As illustrated in Figure 7, FNY inputs do contribute to the ensemble-based influenza prediction estimates.

## Discussion

We have described 3 different participatory surveillance systems, WISDM, Influenzanet, and FNY, and we have shown how modeling and simulation can be or has been combined with participatory disease surveillance to (1) measure the nonresponse bias present in a participatory surveillance sample using WISDM and (2) now-cast and forecast influenza activity in different parts of the world using Influenzanet and FNY.

While the advantages of participatory surveillance, compared to traditional surveillance, include its timeliness, lower costs, and broader reach, it is limited by a lack of control over the characteristics of the population sample. Modeling and simulation can help overcome this limitation.

Use of MTurk and WISDM combined with synthetic population modeling, as shown here, is one way to measure nonresponse and sample bias. The results measure the nonresponse and sample bias for three epidemic outcomes (ie, epidemic size, peak infection rate, and time-to-peak). As shown in Table 1, a lower transmission rate results in a higher nonresponse bias and higher total bias. Total biases for all 3 metrics are statistically significant. Also for all three metrics, the nonresponse bias is larger than the sample bias and dominates the total bias. This is consistent with the fact that MTurk survey responders tend to be younger, educated males among whom the incidence of disease is typically lower than much of the rest of the population. Results for the higher transmission rate are similar. In summary, WISDM-based results show that the bias that occurs in a skewed survey sample can be measured through modeling and simulation to infer more dependable observations than what can be derived from the survey data alone.

Our results confirmed that combining participatory surveillance information from FNY with modeling approaches improve short-term influenza activity predictions. In addition, we described how combining participatory surveillance information with other data sources, by means of a robust machine-learning modeling approach, has led to substantial improvements in short-term influenza activity predictions [49]. Information from participatory surveillance may also help improve influenza forecasting approaches such as those proposed in other studies [53-56].

Moreover, we have shown how by combining digital participatory surveillance data with a realistic data-driven epidemiological model we can provide both short-term now-casts (1 or 2 weeks in advance) of epidemic intensities and long-term (3 or 4 weeks in advance) forecasts of significant indicators of an influenza season. It is indeed the participatory surveillance data component that allows for real-time forecasts of seasonal influenza activity. ILI incidence estimates produced by traditional surveillance systems undergo weekly revisions, are usually released with at least a 1-week lag, and lack the geographical resolution needed to inform high-resolution dynamical models such as GLEAM. Participatory surveillance data are available as soon as participants report their health status. This real-time component allows for accurate now-casting (1 week) and forecasting (2, 3, and 4 weeks) as soon as the influenza activity among the population begins, even before the epidemic curve surpasses the threshold. Data from traditional surveillance up until a specific week are used to fit the selected ensembles which then provide predictions for the upcoming weeks, but these ensembles need to be generated by using the high-resolution real-time data from participatory surveillance.

For future work aimed at harmonizing these three approaches, results from the WISDM platform about nonresponse bias could be used to assess similar biases in groups of self-selected individuals participating in Influenzanet and FNY [24].

The projects described here not only strengthen the case for modeling and simulation becoming an integral component of the epidemic surveillance process, but they also open up several new directions for research. Important questions are yet to be answered. How do we optimally integrate other sources of data with data obtained through participatory surveillance? How do we incorporate participatory surveillance data that are reweighted at each point in time based on active learning techniques to maximize forecast accuracy? How can hypotheses be generated and tested in an abductive setting? An abductive setting is where the models and experiments can be run iteratively to test data-driven hypotheses that evolve as new data arrives in real time.

With the increasing reach of the Internet and cellular communication, participatory surveillance offers the possibility of early detection of and response to infectious disease epidemics. Continued integration of participatory surveillance with modeling and simulation techniques will help to strengthen real-time epidemic science and provide a more rigorous understanding of epidemic conditions.

## Abbreviations

CDC: Centers for Disease Control and Prevention
FNY: Flu Near You
GLEAM: Global Epidemic And Mobility model
ILI: influenza-like illness
ILINet: Influenza-Like Illness Surveillance Network
MAPE: mean absolute percentage error
MIDAS: Models of Infectious Disease Agent Study
MTurk: Mechanical Turk
NIH: National Institutes of Health
NSF: National Science Foundation
SEIR: Susceptible, Exposed, Infected, and Recovered
WISDM: Widely Internet-Sourced Distributed Monitoring
